# Analytical Solution of the Non-Stationary Heat Conduction Problem in Thin-Walled Products during the Additive Manufacturing Process

**DOI:** 10.3390/ma14144049

**Published:** 2021-07-20

**Authors:** Dmitrii Mukin, Ekaterina Valdaytseva, Gleb Turichin

**Affiliations:** Institute of Laser and Welding Technology, Saint Petersburg State Marine Technical University, 190121 St. Petersburg, Russia; valdaitseva@mail.ru (E.V.); gleb@ltc.ru (G.T.)

**Keywords:** additive manufacturing, direct metal deposition, analytical modeling, non-stationary temperature field

## Abstract

The work is devoted to the development of a model for calculating transient quasiperiodic temperature fields arising in the direct deposition process of thin walls with various configurations. The model allows calculating the temperature field, thermal cycles, temperature gradients, and the cooling rate in the wall during the direct deposition process at any time. The temperature field in the deposited wall is determined based on the analytical solution of the non-stationary heat conduction equation for a moving heat source, taking into account heat transfer to the environment. Heat accumulation and temperature change are calculated based on the superposition principle of transient temperature fields resulting from the heat source action at each pass. The proposed method for calculating temperature fields describes the heat-transfer process and heat accumulation in the wall with satisfactory accuracy. This was confirmed by comparisons with experimental thermocouple data. It takes into account the size of the wall and the substrate, the change in power from layer to layer, the pause time between passes, and the heat-source trajectory. In addition, this calculation method is easy to adapt to various additive manufacturing processes that use both laser and arc heat sources.

## 1. Introduction

Additive technologies, in particular direct energy deposition (DED) technologies, are actively developing and are already used in modern production in the manufacture of metal parts and repair and restoration work [1,2,3,4,5]. Laser, electron beam, plasma, and arc are used as the main heat source, and the filler material can be used both in the form of powder and in the form of wire. A wide range of materials can be used: steel, titanium, aluminum, nickel alloys, and composites [6,7,8,9,10,11].

A characteristic feature of the direct deposition process is that the material undergoes multiple heating and cooling processes, including partial remelting of already-formed layers. Such a complex temperature field, which changes both in space and in time, significantly affects the local microstructure and its evolution, residual stresses, and deformations, as well as the distribution of defects, which can significantly affect the mechanical properties, and hence the entire product’s reliability.

The problem of determining transient temperature fields can be investigated using experiments and mathematical modeling. Experimental determination is time-consuming and costly due to the large number of the process operating parameters. Typically, thermocouples and infrared thermography are used to determine temperature fields [12,13,14,15,16]. However, it is not possible to directly measure the object temperature using thermography. The accuracy of determining the surface temperature is limited by the unknown emissivity *ε*. In addition, emissivity can change during additive manufacturing (AM), as it depends on material, temperature, viewing angle, surface roughness, and presence of oxide films [17,18]. As a consequence, the instrument-calibration process is extremely difficult. The use of thermocouples makes it possible to determine the transient temperature much more accurately; however, thermocouples must be thin enough, and at the same time they only allow measuring the temperature locally, even if there are several of them.

Various methods of mathematical modeling are used to determine the three-dimensional temperature fields. Currently, one of the most common methods for calculating transient temperature fields in the AM process is the finite element method [19,20,21]. The objective of such calculations is usually to determine the temperature field evolution, temperature gradients, and their effect on residual stresses, which is related to a thermomechanical problem.

The main difficulty of modeling DED processes for large products is associated with the large temporal and spatial domain of the calculation. To determine the most accurate transient temperature fields, the time step should be microseconds, while the total deposition time is many hours. The melt pool has a characteristic size of the order of a few millimeters, while the model of the entire product is usually on a meter or sub-meter scale. As a consequence, such calculations require enormous computing power and time.

There are several approaches to solve such problems. The first approach assumes minimal time steps and a fine mesh of the part, which leads to fairly accurate temperature values, but the time spent on computations can be huge [22,23]. The second approach assumes a scheme according to which the material is added either in parts of a layer (a hatch-by-hatch), or in whole layers at once (layer-by-layer), or in several layers at once [21,24]. In this case, the deposited energy for a period of time corresponding to the trajectory traversed is distributed throughout the added material. Thus, the simulation time can be significantly reduced. As a result, only the history of the average temperature is recorded, but not the local thermal history.

In some works, the finite volume method is used to determine transient temperature fields, which also takes into account convective heat transfer. Due to the complexity of such calculations, the simulated samples are bodies with characteristic dimensions of the order of several tens of millimeters [25,26,27]. As already mentioned, real products have dimensions of the order of a meter, which ensures that the process conditions are different from the simulated ones, in particular, the temperature, cooling rates, and temperature gradients will differ.

In comparison with the above models, the peculiarity of this work is to provide a fast, simple, and universal, but at the same time reliable, analytical method for calculating transient temperature fields in the AM process. Maintaining stable temperatures and melt-pool sizes is one of the key means of controlling the process stability. Then, as a result of using this calculation method, it becomes possible to theoretically determine the influence of the mode parameters on the formation of the deposited layers, as well as to select stable process modes. In addition, the application of this method will make it possible to study the degree of influence of local quasiperiodic temperature fields, which always accompany direct deposition processes, on structural phase transformations.

## 2. Model and Methods Description

### 2.1. Problem Statement

The following physical assumptions were made:

- The physical properties of the substrate and the filler material (specific heat capacity *c*, density *ρ*, thermal conductivity *λ*, thermal diffusivity *a*) are temperature-independent.
- The effect of convection of liquid metal is not considered.
- Heat flux distribution of the heat source *q_h_* is presented as a surface normally distributed heat source.
- Heat transfer occurs according to Newton’s law.

In order to obtain the temperature field in the substrate and the deposited layers, it is necessary to solve the following linear non-stationary heat-conduction problem in a Cartesian coordinate system *x*, *y*, *z*:

(1) $$\frac{\lambda }{c\rho }\left(\frac{\partial^{2}T}{\partial x^{2}}+\frac{\partial^{2}T}{\partial y^{2}}+\frac{\partial^{2}T}{\partial z^{2}}\right)=\frac{\partial T}{\partial t},$$

where *λ*—thermal conductivity of the material, *c*—specific heat capacity, and *ρ*—density.

The initial temperature of the substrate is equal to the ambient temperature:

(2) $${T(x,y,z,t)|}_{t=0}=T_{0}.$$

Boundary conditions on the front surface of the computational domain:

(3) $$\;-\lambda \frac{\partial T}{\partial n}=q_{h}(x,y),$$

where *q_h_* (*x*, *y*) is the heat flux density.

The adiabatic boundary is set on other surfaces where there are no heat sources.

Heating of a product in the AM process is described as the action of a surface elliptical heat source with a power density *q_h_* (*x*, *y*). In the *x0y* plane, the power-density distribution is described by the Gaussian function:

(4) $$q_{h}(x,y)=\frac{Q_{h}\cdot \eta }{\pi \cdot R_{H}^{2}}\sin\beta \cdot \exp\left(-\frac{{\left(x\;\;\sin\beta \right)}^{2}+y^{2}}{R_{H}^{2}}\right),$$

where *Q_h_*—heat-source power, *η*—heat efficiency, *R_H_*—effective radius of the heat source, and *β*—tilt angle.

### 2.2. Analytical Model of Non-Stationary Heat Transfer

The temperature increment at an arbitrary point with coordinates *x*, *y*, *z* (in a fixed coordinate system) at any time *t* from an elementary point source that acted at time *t*’ on the surface of a semi-infinite body is known and is equal to *dT* [28]:

(5) $$dT(x,y,z,t,t^{\prime })=\frac{2\;q\;dt^{\prime }}{c\rho {\left[4\pi a(t-t^{\prime })\right]}^{3/2}}\exp\left(-\frac{{\left[x-vt^{\prime }\right]}^{2}+y^{2}+z^{2}}{4a(t-t^{\prime })}\right),$$

where *q*—point heat-source power, *v*—heat-source moving speed (cladding speed), and *a*—thermal diffusivity, equal to *a* = *λ*/(*c*·*ρ*).

When calculating the repeated heating and cooling process of thin-walled products, it is impossible to neglect heat transfer to the environment, since it leads to a noticeable error in determining the temperature. The smaller the deposited wall thickness, the greater the heat-transfer effect to the environment. Considering the above, it is necessary to obtain a non-stationary equation for the heat-propagation process, taking into account heat transfer to the air.

Let it be supposed that the heat transfer occurs according to Newton’s law, but the temperature along the thickness is equalized instantly. Then the heat transfer from the wall side surfaces is taken into account by introducing the multiplier *e*^−*b*(*t*−*t′*)^ into Equation (5). It means only a decrease in the average temperature in the section, but does not consider the temperature unevenness along the wall thickness. Thus, heat transfer is equivalent to a volumetric heat sink, while the condition of the adiabatic boundary is still satisfied. Then Equation (5) takes the form:

(6) $$dT(x,y,z,t,t^{\prime })=\frac{2\;q\;dt^{\prime }}{c\rho {\left[4\pi a(t-t^{\prime })\right]}^{3/2}}\exp\left(-\frac{{\left[x-vt^{\prime }\right]}^{2}+y^{2}+z^{2}}{4a(t-t^{\prime })}-b(t-t^{\prime })\right),$$

where $b=\frac{2\alpha }{c\rho h}$—coefficient of heat loss, *α*—coefficients of surface heat transfer, and *h*—wall thickness.

A moving heat source can be represented as elementary instantaneous sources acting sequentially and displaced relative to each other. Let us sum up the temperature increments from all elementary sources that acted in the general case during the time from *t*_1_ to *t*_2_ and make elementary transformations:

(7) $$\Delta T(x,y,z,t,t_{1},t_{2})=\frac{2\;q}{c\rho {\left(4\pi a\right)}^{3/2}}\exp\left(-\frac{v(x-vt)}{2a}\right)\int_{t1}^{t2}\exp\left(-\left[\frac{v^{2}\;}{4a}+b\right](t-t^{\prime })-\frac{R{\left(x,y,z\right)}^{2}}{4a(t-t^{\prime })}\right)\frac{dt^{\prime }}{{\left(t-t^{\prime }\right)}^{3/2}},$$

where $R(x,y,z)=\sqrt{{\left(x-\nu t\right)}^{2}+y^{2}+z^{2}}$—distance from the heat source to the considered point of the body, *t*—considered the moment in time, *t*_1_—the start time of the source action, *t*_2_—the time when the source ends its action, and *t* > *t*_2_ ≥ *t*_1_ ≥0.

A sufficient condition for determining the temperature field when the source has not yet stopped its action (when *t* = *t*_2_) is the difference between *t* and *t*_2_ by an infinitesimal value *o*(*t*).

To obtain the temperature field at any time ∆*T* (*x*, *y*, *z*, *t*, *t*_1_, *t*_2_), it is necessary to calculate the integral in Equation (7) with the limits of integration *t*_1_ and *t*_2_. For this, the integral in Equation (7) is represented as the difference of two integrals. Then the solution for a moving point source can be obtained using the substitution $u^{2}=1/\sqrt{t-t^{\prime }}$ and the known integral 1.3.3.20 [29]:

(8) $$\begin{array}{l} \Delta T(x,y,z,t,t_{1},t_{2})=\frac{2\;q}{4\pi \lambda R\;}\frac{1}{2}\exp\left(-\frac{v\left(x-vt\right)}{2a}\right)\cdot \\ \cdot \{\;[\exp\left(-\frac{RvB}{2a}\right)\cdot \Phi^{*}\left(\frac{-R}{2\sqrt{a(t-t_{2})}}+\frac{vB\sqrt{\left(t-t_{2}\right)}}{2\sqrt{a}}\right)-\exp\left(\frac{RvB}{2a}\right)\Phi^{*}\left(\frac{R}{2\sqrt{a(t-t_{2})}}+\frac{vB\sqrt{\left(t-t_{2}\right)}}{2\sqrt{a}}\right)]-\\ -[\exp\left(-\frac{RvB}{2a}\right)\cdot \Phi^{*}\left(\frac{-R}{2\sqrt{a(t-t_{1})}}+\frac{vB\sqrt{\left(t-t_{1}\right)}}{2\sqrt{a}}\right)-\exp\left(\frac{RvB}{2a}\right)\Phi^{*}\left(\frac{R}{2\sqrt{a(t-t_{1})}}+\frac{vB\sqrt{\left(t-t_{1}\right)}}{2\sqrt{a}}\right)]\;\}, \end{array}$$

where $R=\sqrt{{\left(x-v\;t\right)}^{2}+y^{2}+z^{2}}$, $B=\sqrt{1+\frac{4ba}{v^{2}}}$, $\Phi^{*}(u)=1-\frac{2}{\sqrt{\pi }}\int_{0}^{u}e^{-u^{2}}du$.

**Figure 1:**
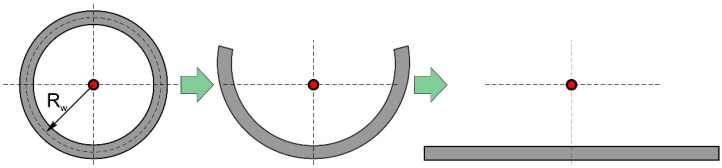
Scheme of unwrapping of a closed wall (cylindrical wall).

**Figure 2:**
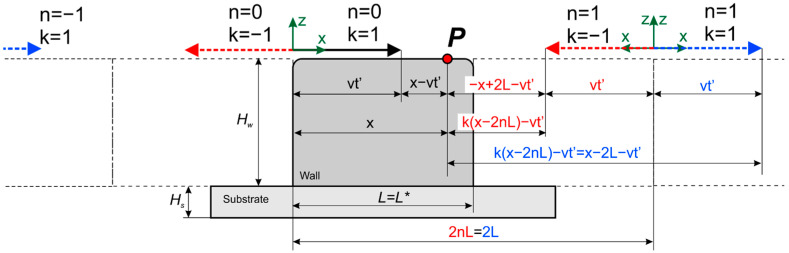
Scheme of introducing imaginary sources to consider the effect of the limited wall size for a single wall.

**Figure 3:**
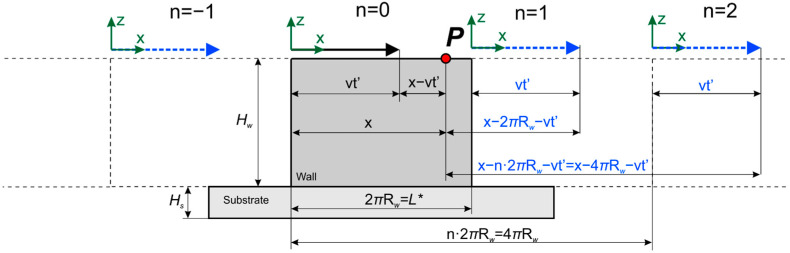
Scheme of introducing imaginary sources to consider the effect of the limited wall size for a closed wall.

Let us now consider the effect of the limited wall size under the assumption that its boundaries are adiabatic. This assumption allows the use of the method of images. To do this, it is necessary to mirror the actual heat source and each mirrored source from the planes *x* = 0, *x* = L*, where L* is the wall length, from the planes *z* = 0 and *z* = H, where H is the wall height (*H* = *H_s_* + *H_w_*), and also from the side wall boundaries *y* = *W*/2, *y* = −*W*/2, where *W* is the wall width. As a result, we obtain a system of an infinite number of heat sources. A cylindrical wall (generally a closed wall) can be represented as a single wall by unwrapping the wall around one of its generatrices (Figure 1). Figure 2 and Figure 3 show the schematic of the reflection of sources along the *x*-axis for a single wall and a closed single wall, respectively. The red color denotes imaginary sources for which *k* = 1, and the blue color denotes that *k* = 1. The temperature field is calculated at an arbitrary point *p*.

Then, the temperature field in a bounded wall is determined by the following sum of the corresponding solutions for an unbounded semi-infinite body:

(9) $$\begin{array}{ll} dT(x,y,z,t\;,t_{1\;},t_{2\;})=\sum_{p=-\infty }^{+\infty }\; & \sum_{j=-\infty }^{+\infty }\;\sum_{n=-\infty }^{+\infty }\;\sum_{k}^{}\;\int_{t1}^{t2}\frac{2\;q\;dt^{\prime }}{c\rho {\left[4\pi a(t-t^{\prime })\right]}^{3/2}}\cdot \\ & \cdot \exp\left(-\;\frac{{\left[X-vt^{\prime }\right]}^{2}+{\left(y+jW\right)}^{2}+{\left(z+2pH\right)}^{2}}{4a(t-t^{\prime })}-b(t-t^{\prime })\right), \end{array}$$

where $X=k(x-2nL^{*})$—for the case of a single wall; $X=k(x-nL^{*})$—for the case of a closed wall; *k* = −1, 1—for the case of a single wall; *k* = 1—for the case of a closed wall; L* = *L*—for the case of a single wall and L* = *2*π*R*_w_—for the case of a closed wall. Summation over *k* and *n* considers the limited length; while for summation over *j* and *p,* over width and height, respectively.

Let us integrate Equation (9) from *t*_1_ to *t*_2_, repeating all the steps that have been done to obtain Equation (8) and take into account that the source can be distributed over the surface of the computational domain. The result is the equation:

(10) $$\begin{array}{l} dT(x,y,z,xs,ys,t,t_{1},t_{2})=\sum_{p=-\infty }^{+\infty }\;\sum_{j=-\infty }^{+\infty }\;\sum_{n=-\infty }^{+\infty }\sum_{k}^{}\frac{2\;q(xs,ys)dxs\;dys}{4\pi \lambda R\;}\frac{1}{2}\exp\left(-\frac{v\left(X-vt-xs\right)}{2a}\right)\cdot \\ \cdot \;\{\;[\exp\left(-\;\frac{RvB}{2a}\right)\cdot \Phi^{*}\left(\frac{-R}{2\sqrt{a(t-t_{2})}}+\frac{vB\sqrt{\left(t-t_{2}\right)}}{2\sqrt{a}}\right)-\exp\left(\frac{RvB}{2a}\right)\cdot \Phi^{*}\left(\frac{R}{2\sqrt{a(t-t_{2})}}+\frac{vB\sqrt{\left(t-t_{2}\right)}}{2\sqrt{a}}\right)]-\\ -[\exp\left(-\;\frac{RvB}{2a}\right)\cdot \Phi^{*}\left(\frac{-R}{2\sqrt{a(t-t_{1})}}+\frac{vB\sqrt{\left(t-t_{1}\right)}}{2\sqrt{a}}\right)-\exp\left(\frac{RvB}{2a}\right)\cdot \Phi^{*}\left(\frac{R}{2\sqrt{a(t-t_{1})}}+\frac{vB\sqrt{\left(t-t_{1}\right)}}{2\sqrt{a}}\right)]\;\}, \end{array}$$

where $R=\sqrt{{\left(X-vt-xs\right)}^{2}+{\left(y+jW-ys\right)}^{2}+{\left(z+2pH\right)}^{2}}$; and *xs*, *ys* are the *x*, *y* coordinates, respectively, of the point source in the coordinate system associated with the source.

By shifting the origin for each pass in the AM process, it is possible to set the times *t*_1_ and *t*_2_ in such a way that *t*_1_ = 0 always, and $t_{2}^{}=\;\left\{ \begin{array}{ll} t-o(t), & if\;t\leq \frac{L^{*}}{v};\\ \frac{L^{*}}{v}, & if\;t>\frac{L^{*}}{v}; \end{array}\right.$. In this case, *t*_1_ and *t*_2_ are not arguments to the *dT* function.

When using a distributed heat source, it is necessary to integrate Equation (10) over the source area (the area radius is equal to *R_b_*). Then the heating temperature ∆*T_preh_* (*x*, *y*, *z*, *t*, *t*_1_, *t*_2_) can be obtained as:

(11) $$\Delta T_{preh}(x,y,z,t,t_{1},t_{2})=\int_{-W/2}^{+W/2}\;\int_{-Rb}^{+Rb}dT(x,y,z,xs,ys,t,t_{1},t_{2}).$$

The function series in Equation (10) generally converge rapidly, so in practice, it is possible to limit the series to the first few terms. However, the longer the considered heating or cooling time *t* and/or thermal diffusivity *a*, the greater the number of series terms must be considered. In other words, the number of series terms is directly proportional to $\sqrt{4at}$. The criterion for choosing the number of series terms is the fact that the tangent of the slope of the tangent line to the temperature-distribution curve along the normal to the surfaces is equal to 0 at the adiabatic boundary, which corresponds to an angle of 0°.

It should be noted that the practical application of Equation (10) is not convenient, since *R* can have a large value in absolute value, then in the product $\exp\left(\frac{RvB}{2a}\right)\cdot \Phi^{*}\left(\frac{R}{2\sqrt{a(t-t_{1,2})}}+\frac{vB\sqrt{t-t_{1,2}}}{2\sqrt{a}}\right)$, the multiplier $\exp\left(\frac{RvB}{2a}\right)$ tends toward a large value, which is taken as infinity (infinitely large value) when using mathematical and computational software. The multiplier $\Phi^{*}\left(\frac{R}{2\sqrt{a(t-t_{1,2})}}+\frac{vB\sqrt{t-t_{1,2}}}{2\sqrt{a}}\right)$ tends toward 0 (infinitesimal value). Consequently, an indeterminate form of the type (0 × ∞) arises in Equation (10).

For the evaluation of the indeterminate form, let us use, for example, the known approximation of the error function erf (*x*) using elementary functions 7.1.26 [30]:

(12) $$\mathrm{erf}(x)=1-\left(a_{1}k+a_{2}k^{2}+\ldots +a_{5}k^{5}\right)e^{-x^{2}}+\varepsilon (x),$$

where $k=\frac{1}{1+px}$, $\left|\varepsilon (x)\right|\leq 1.5\cdot 10^{-7}$, $x=\frac{R}{2\sqrt{a(t-t_{1,2})}}+\frac{vB\sqrt{t-t_{1,2}}}{2\sqrt{a}}$, *p* = 0.3275911, *a*_1_ = 0.254829592, *a*_2_ = −0.284496736, *a*_3_ = 1.421413741, *a*_4_ = −1.453152027, and *a*_5_ = 1.061405429.

Using Equations (10) and (12) and performing elementary transformations, it is easy to get rid of the indeterminate form.

### 2.3. Influence of the Substrate on the Temperature Field

The next step is to consider the effect of the substrate and the wall height on the deposited wall temperature. The conditions for the formation of the layers differ as to their number increases. It is primarily due to the different heat-removal conditions caused by the heat accumulation in the wall and substrate during the initial deposition period and by an increase in the wall temperature. Upon reaching a steady state of the deposition process, the wall temperature stops rising.

It is convenient to use Green’s function method to calculate the temperature field for bodies with a simple geometric shape. The cross-section of the wall deposited on the substrate has a T-shape, which complicates the use of this method.

**Figure 4:**
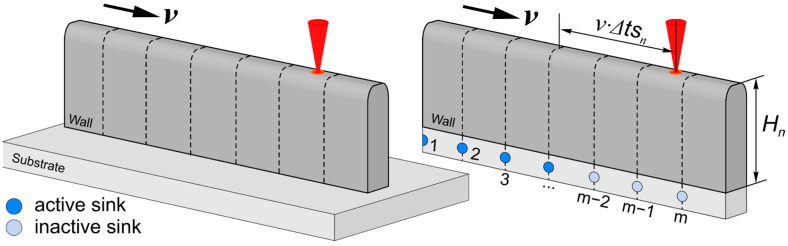
Scheme of the introduction of sinks to consider the influence of the substrate in the AM process (the drains are shown for the current passage).

In this regard, an equivalent computation scheme for calculating temperature fields was considered. Imagine a wall on a substrate as just a wall, removing the side parts of the substrate (Figure 4). To take into account the removed mass of the substrate, let us introduce heat sinks with certain energy equal to that accumulated in the substrate during deposition. These fixed sinks are activated as the real heat source moves, thereby the introduced sinks simulate the presence of a substrate.

The temperature increment in the equilibrium state after the next pass is:

(13) $$\Delta T=\frac{Q}{cm}=\frac{Q_{h}\;\eta \;\frac{L^{*}}{v}}{c\rho V_{s}+c\rho V_{w}},$$

where *Vs*—the substrate volume, and *V_w_*—the wall volume.

To find the energy of the sinks, it is necessary that the increment in the wall temperature in the absence of a substrate corresponds to the increment in the wall temperature in the presence of a substrate. Thus, the total energy of the sinks in the *n*th passage is determined from the expression:

(14) $$E_{n}=Q_{h}\;\eta \frac{L^{*}}{v}\left(1-\frac{c\rho {V_{s}}^{\prime }+c\rho V_{w}(n)}{c\rho V_{s}+c\rho V_{w}(n)}\right),$$

where *V_s_*′—truncated substrate volume, and *V_w_* (*n*)—wall volume on the *n*th passage.

To find the power of each fixed sink, it is necessary to know the time of their action *ts_n_* and the total energy of all sinks *E_n_*, which is already known. The action time of the sinks is proportional to the distribution time of uneven temperature; that is, $ts_{n}\sim \frac{R_{n}{}^{2}}{4a}$, where *R_n_* is the characteristic size of the deposition wall after *n* passes. So, the power of each sink is:

(15) $$q_{s}^{n}=\frac{E_{n}^{}}{2\cdot \sum_{i=0}^{m-1}\left(ts_{n}-\frac{i}{m}\frac{L^{*}}{v}\right)},$$

where *m*—number of sinks.

The total energy of the sinks decreases with an increase in the number of layers, and the action time increases, which indicates a decrease in the influence of the substrate as the wall height increases.

To take into account the inertia of the heat-propagation process, the sinks act with a delay $\Delta ts_{n}$ relative to the real heat-source action (see Figure 4), while $\Delta ts_{n}\sim \frac{H_{n}^{2}}{4a}$.

The equation describing the temperature field created by fixed-point sinks on the surface of a semi-infinite body can be obtained by setting *v* = 0 in Equation (8). Then, the equation describing heat propagation, considering the limited wall size, has the form:

(16) $$\begin{array}{l} \Delta T_{sk}(x,y,z,t)=\sum_{u=-1,1}^{}\;\sum_{p=-\infty }^{+\infty }\;\sum_{j=-\infty }^{+\infty }\;\sum_{n=-\infty }^{+\infty }\;\sum_{k}^{}\;\sum_{i=0}^{m-1}\;\frac{2\;q}{4\pi \lambda R\;}\frac{1}{2}\cdot \\ \cdot \{\;[\exp\left(-\frac{R\sqrt{b}}{\sqrt{a}}\right)\cdot \Phi^{*}\left(\frac{-R+\sqrt{4ba}(t-t_{2}^{i})}{2\sqrt{a(t-t_{2}^{i})}}\right)-\exp\left(\frac{R\sqrt{b}}{\sqrt{a}}\right)\Phi^{*}\left(\frac{R+\sqrt{4ba}(t-t_{2}^{i})}{2\sqrt{a(t-t_{2}^{i})}}\right)]-\\ -[\exp\left(-\frac{R\sqrt{b}}{\sqrt{a}}\right)\cdot \Phi^{*}\left(\frac{-R+\sqrt{4ba}(t-t_{1}^{i})}{2\sqrt{a(t-t_{1}^{i})}}\right)-\exp\left(\frac{R\sqrt{b}}{\sqrt{a}}\right)\Phi^{*}\left(\frac{R+\sqrt{4ba}(t-t_{1}^{i})}{2\sqrt{a(t-t_{1}^{i})}}\right)]\;\}, \end{array}$$

where $R=\sqrt{{\left(k(x-2nL^{*})-\left[\frac{i}{m}L^{*}\right]\right)}^{2}+{\left(y-\frac{W}{2}+jW\right)}^{2}+{\left(z-u\left(H-\frac{H_{s}}{2}\right)+2pH\right)}^{2}}$,

$t_{1}^{i}=\;\left\{ \begin{array}{ll} t-o(t), & if\;t\leq \frac{i}{m}\frac{L^{*}}{v}+\Delta ts;\\ \frac{i}{m}\frac{L^{*}}{v}+\Delta ts, & if\;t>\frac{i}{m}\frac{L^{*}}{v}+\Delta ts; \end{array}\right.$ $t_{2}^{i}=\;\left\{ \begin{array}{ll} t-o(t), & if\;t\leq ts+\Delta ts;\\ ts+\Delta ts, & if\;t>ts+\Delta ts; \end{array}\right.$

In Equation (16), the summation over *i* is additionally introduced, which considers the action of each activated sink at the considered moment of time.

Taking into account the linearity of the thermal problem, the temperature field of heating on the *n*th layer *T_n_* is presented as the sum of the temperature fields as a result of the heat-source action and the heat-sink action at each pass.

In this work, the temperature field *T_n_* after the deposition of the *n*th number of layers, taking into account the linearity of the thermal problem, is represented as a temperature field as a result of the heat-source action depositing the *n*th layer, in front of which are *n*−1 heat sources, and also the action of sinks. These heat sources and sinks have equal or different power and operate at equal or different intervals of time, depending on the deposition strategy or scheme. Then, the heating temperature can be calculated using the following equation:

(17) $$T_{n}(x,y,z,t)=T_{0}+\sum_{i=0}^{n-1}\;[\Delta T_{preh}\left(x,y,z,t+i\left[\frac{L^{*}}{v}+t_{pause}\right]\right)\;-\Delta T_{sk}\left(x,y,z,t+i\left[\frac{L^{*}}{v}+t_{pause}\right]\right)\;],$$

where *t_pause_*—the pause time between passes, index *i* = 0 corresponds to the last pass, and index *i* = *n* − 1 corresponds to the first pass.

## 3. Results and Discussions

To verify the developed non-stationary heat-transfer model, two deposition processes were chosen and simulated. The first process was the DLD (direct laser deposition) process of a thin wall using Ti-6Al-4V powder [31], and the second process was the WAAM (wire arc additive manufacturing) process of a cylindrical thin wall using H08Mn2Si steel wire [32]. Type K thermocouples were used to measure the temperature. In the case of the DLD, the thermocouple was located on the substrate surface opposite the center of the wall at a distance of 0.5 mm. In the case of WAAM, the thermocouple was located on the substrate surface at a distance of 3 mm from the cylindrical wall. Both experiments were accompanied by calculations using numerical methods. The parameters of the modes are presented in Table 1. Details of the conditions for setting up experiments, placement of thermocouples, simulation parameters, and the values of thermophysical properties are described in detail in the corresponding works. It is worth mentioning that in the simulation in this work, the values of properties were adopted as constant, and averaged in the temperature interval between the ambient temperature and the melting point.

**Figure 5:**
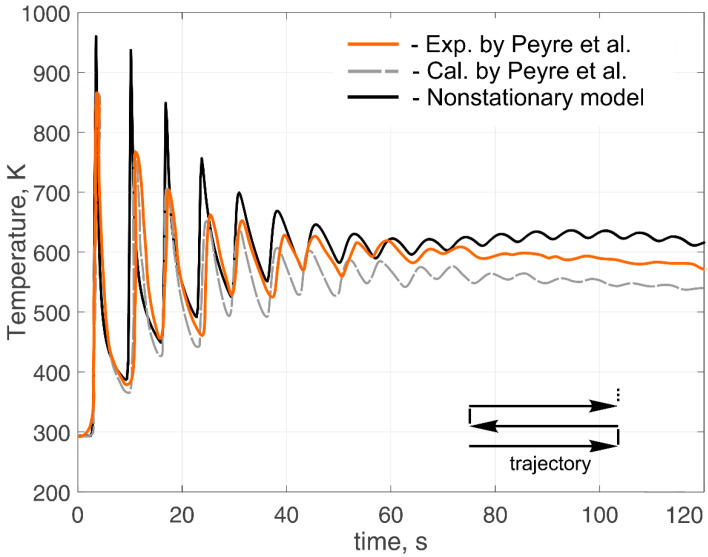
Comparison of calculated and experimental thermal cycles during deposition of the single wall of 20 layers (*t_pause_* = 0 s). Experimental results obtained by Peyre [31].

**Figure 6:**
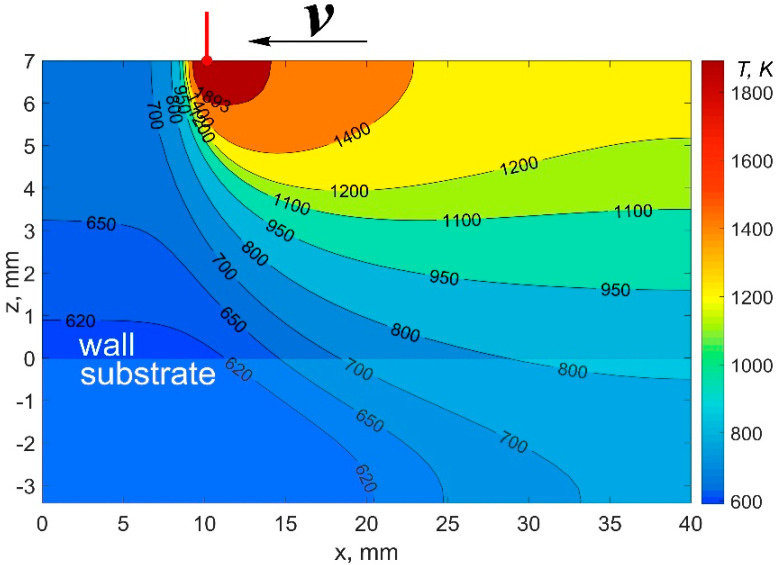
Calculated temperature field of the deposited wall in the longitudinal section during cladding of the 20th layer.

**Figure 7:**
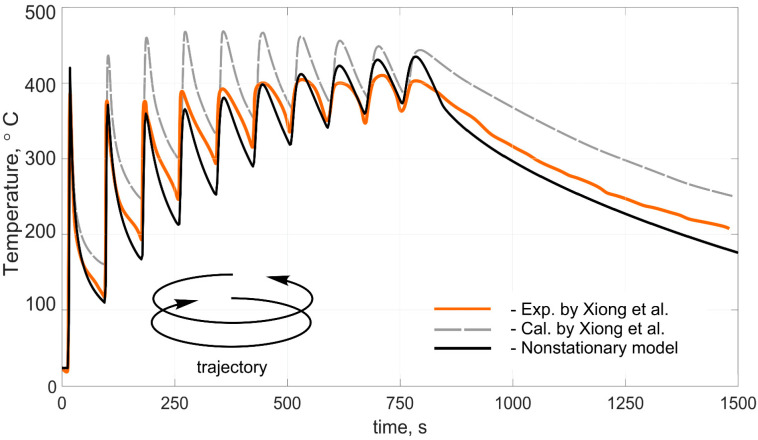
Comparison of calculated and experimental thermal cycles during deposition of the cylindrical wall of 10 layers (*t_pause_* = 33 s). Experimental results obtained by Xiong [32].

Figure 5 shows the compared results of the experimental data of temperature, as well as the data obtained using numerical methods with the calculations performed according to this work. Figure 6 shows the calculated temperature distribution in the deposited wall in its longitudinal section when the 20th layer was cladded. Figure 7 shows the comparing results of the experimental data of cylindrical wall temperature, as well as the data obtained using numerical methods with the calculations made according to this work.

The calculated transient temperature fields agreed satisfactorily with the experimental results both in the heating phase and in the free-cooling phase. In Figure 5, the calculated curve shows an increased temperature-change amplitude in comparison with the experimental data after 75 s. This can be explained by the fact that the experimental sample had a more complex cross-sectional shape, which allowed the heat flux to diverge in all directions at the junction of the wall and the substrate. Thus, it led to a decrease in the temperature change amplitude in the region below the plane of junction between the wall and the substrate. When measuring the wall temperature directly, this effect did not appear. It is worth mentioning that the calculations were performed under cardinally different conditions; namely, the heat sources, the process parameters, the movement trajectory, and the pause time were different. This fact suggests that the developed model made it possible to reproduce temperature fields in a wide range of technological process parameters and for various product configurations. Ignoring heat transfer to the environment greatly distorts the temperature, and given the specificity of additive manufacturing technologies in the form of multiple heating and cooling processes, it leads to a significant error. In addition, this method made it possible to calculate temperature fields in times in the order of several minutes using a personal computer. For this reason, this calculation method can be used to predict the temperature of large parts.

## 4. Conclusions

The three-dimensional, non-stationary heat-transfer model was developed for the direct deposition processes of thin-walled parts with various configurations. The calculated transient temperature fields obtained using the developed model agreed satisfactorily with the experimental results, both in the heating phase and in the free-cooling phase. At the same time, the presented calculations were performed with cardinally different input parameters of the model; namely, the types of heat sources, the process technological parameters, thermophysical properties of materials, movement trajectories, and pause times were different. These facts suggest that the model allowed reproducing three-dimensional temperature fields in a wide range of technological process parameters and for various product configurations.

To develop the model, a three-dimensional analytical solution of the non-stationary heat-conduction equation for a moving distributed heat source was obtained, taking into account heat transfer to the environment. The model made it possible to calculate all the temperature field characteristics; in particular, thermal cycles, temperature gradients, and the cooling rate in a path during the deposition process at any time. In this case, the wall and the substrate sizes, the change in power from layer to layer, the pause time between passes, and the trajectory of the heat source were taken into account.

Thus, the developed model can be used to predict temperature fields, as well as to study the degree of influence of local quasiperiodic temperature fields, which always accompany direct deposition processes, on structural phase transformations.

The proposed calculation scheme applies to thin-walled products; however, it can be extended for the case of multi-pass thick walls. In addition, this model can be easily adapted to various direct deposition technologies that use a laser, electron beam, plasma, or arc as the main heat source.

## Figures and Tables

**Table 1:** Deposition-mode parameters.

Process	Heat Source	Power (W)	Cladding Speed (mm·s^−1^)	Heat Convection (W·K^−1^·m^−2^)	Heat Efficiency	Pause Time(s)
DLD	laser	600	6	20	0.35	0
WAAM	arc	2850	5	5.7	0.85	33

## Data Availability

Not applicable.
